# Renal Angiomyolipoma With Caval Extension and Pulmonary Fat Embolism: A Case Report

**DOI:** 10.1097/MD.0000000000001078

**Published:** 2015-08-07

**Authors:** Suleyman Utku Celik, Akin Firat Kocaay, Yusuf Sevim, Omer Arda Cetinkaya, Ebru Dusunceli Atman, Iskender Alacayir

**Affiliations:** From the Ankara University School of Medicine, Department of General Surgery, Ankara, Turkey (SUC, AFK, OAC, IA); Kayseri Training and Research Hospital, Department of General Surgery, Kayseri, Turkey (YS); and Ankara University School of Medicine, Department of Radiology, Ankara, Turkey (EDA).

## Abstract

Renal angiomyolipoma (AML) is a rare benign tumor of the kidney. Occasionally, it may extend into the renal vein or the inferior vena cava (IVC), but so far of pulmonary embolism in patients with renal AML was rarely reported. Here, a case of symptomatic pulmonary embolism secondary to AML that was placed IVC filter before the operation and then treated with radical nephrectomy is reported.

This case highlights the rare possibility of renal vein and IVC involvement with symptomatic pulmonary fat embolism in renal AML, which may potentially result in fatal complications if not appropriately and cautiously managed with surgical intervention.

## INTRODUCTION

Renal angiomyolipoma (AML), a PEComa, is the most common mesenchymal tumor of the kidney comprised of mature adipose tissue, dysmorphic blood vessels, and smooth muscle.^1,2^ This unusual benign tumor is found in 0.1% to 0.3% of the general population with 2 distinct groups: classic triphasic AML and monotypic epithelioid AML.^1^ The mean age at presentation is 43 years, with a female predominance (male/female ratio of 4:11).^3^

About 80% of AMLs are sporadic and the others associated with genetic syndrome such as tuberous sclerosis and lymphangioleiomyomatosis.^4^ Most patients with sporadic AML are without symptoms, and AMLs are incidentally detected on cross-sectional abdominal imaging that is obtained for an unrelated reason.^5^

AML with malignant character are not often. Very few cases indicate potential malignant behaviors, including involvement of the regional lymph nodes and renal vein or inferior vena cava (IVC) invasion.^6^ However, rarely the renal AML may show intracardiac extension or pulmonary embolism.^7^ Herein, we report a case of lipomatous AML that demonstrates an unusual aggressive behavior with extension into the renal vein, IVC, and symptomatic pulmonary fat embolism simultaneously.

## CASE PRESENTATION

A 33-year-old woman, without any previous medical history, admitted with shortness of breath, chest pain, and tachypnea to an emergency department. On admission, the patient was dyspneic, tachycardic (105/minute) with normal sinus rhythm and normotensive (120/75 mm Hg). Respiratory rate was 19 breaths per min and the lungs were clear on auscultation. Physical examination revealed no significant abnormality. All routine blood tests, electrocardiogram and chest X-ray were also normal. d-dimer level was found as elevated: 1370 ng/mL (0–500).

**FIGURE 1:**
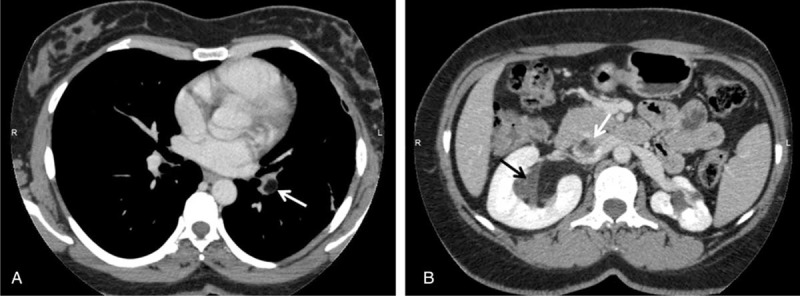
(A) Filling defect (arrow) compatible with fat embolus in the branch of left pulmonary artery is shown on axial CT image. (B) A fat-density lesion in the right renal pelvis (black arrow) extending into the inferior vena cava (white arrow) is demonstrated on axial contrast-enhanced CT image.

Computed tomography (CT) scan of the chest revealed a large pulmonary embolus of fat density within the left lower lobe pulmonary artery (Figure 1A). So, low molecular weight heparin was started and workup done for the possible sources. Doppler ultrasounds of lower and upper extremities ruled out deep vein thrombosis. Antinuclear factor, lupus anticoagulant, anticardiolipin antibodies, thrombophilia parameters, and tumor markers such as CEA, CA 125, CA 19-9, and CA 15-3 were also negative. Contrast-enhanced CT scan of the chest/abdomen/pelvis confirmed the presence of a 56 mm× 40 mm predominantly fat-containing right renal mass with extension into the right renal vein and IVC (Figure 1B). It was resulted in fat embolization in the left pulmonary artery. The thrombus in the pulmonary artery was mainly composed of mature adipose tissue.

**FIGURE 2:**
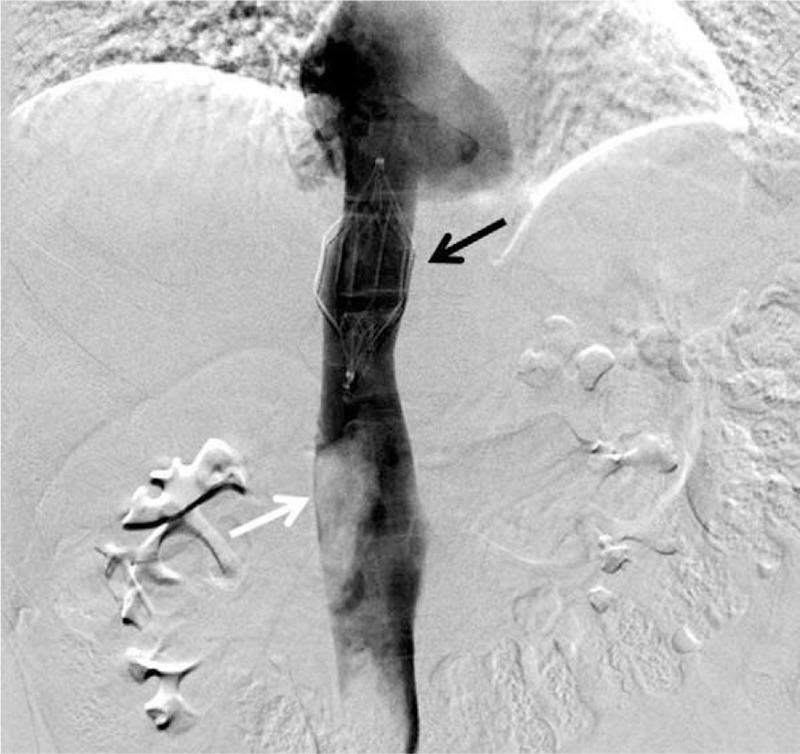
Catheter venography image shows an inferior vena cava filter (black arrow) distal to the large filling defect (tumor thrombus, white arrow) in the inferior vena cava.

In the light of preoperative findings, before the surgery, a temporary IVC filter was placed in the suprarenal position via a right femoral approach to prevent pulmonary complication such as tumor thrombus during nephrectomy. After placing the filter in the IVC (Figure 2), the patient had undergone an open right radical nephrectomy with midline transperitoneal approach. Intraoperatively, the tumor was found to be too deep seated in the renal sinus and a partial nephrectomy could not be performed. During surgery, no enlarged lymph nodes were identified. The patient was discharged by removing the temporary IVC filter on postoperative day 10.

Pathological analysis demonstrated a renal AML (50 mm × 41 mm × 32 mm) in the upper pole of the right kidney with renal vein and IVC involvement. The mass and thrombus were contiguous on gross pathology, and thrombus consistent with AML on micropathology. At 2 months after surgery, an abdominal ultrasound showed no evidence of tumor recurrence or thrombus within the IVC.

## DISCUSSION

Renal AML is a benign mesenchymal tumor of the kidney and composed adipose tissue, vascular elements, and smooth muscle.^1,8^ The term AML was first used by Morgan et al in 1951 and the renal lesion that histologically corresponds to a renal AML was first described by Grawitz in 1900.^7^ AMLs are most commonly sporadic (80%) and a total of 20% are associated with tuberous sclerosis complex or lymphangioleiomyomatosis. Sporadic variety is usually slow-growing, solitary lesions and mostly appears in middle-aged females.^5^ Renal AML is divided into 2 major histologic types: classic and epithelioid. Most patients with sporadic AML have the classic type.^9^ In contrast to the uniformly benign prognosis of classic renal AMLs, epithelioid variants exhibit more aggressive behavior in clinical manifestation, such as extending into the collecting system, renal vein, or IVC and right atrium, although this is infrequent. Malignant transformation is manifested by local recurrence and/or distal metastases.^10^

Most patients are without symptoms, and often incidentally detected on radiological imaging with characteristic sonographic, CT, and magnetic resonance imaging (MRI) findings.^11^ AML more commonly becomes symptomatic in lesions >4 cm, and can present with fever, flank pain, hematuria, gastrointestinal upset, palpable renal mass, renal failure, hypertension, anemia, and shock from retroperitoneal hemorrhage (Wunderlich syndrome).^12^ Biopsy is rarely needed in view of the typical imaging findings of AML.^11^

AMLs typically present as benign lesions, but rarely, extend into the renal veins, IVC or up to the right atrium and invade lymph nodes.^8,13^ However, the renal AML may show intracardiac extension or pulmonary embolism infrequently.^7^ The first case of renal AML presenting with involvement of the IVC was reported by Kutcher et al in 1982.^5,13^ However, so far a literature review by Que et al showed that there are about 45 similar cases have been reported between 1982 and 2013.^10^

The indications for treatment, though somewhat controversial; the most studies recommend surgical treatment for large tumors (≥4 cm), even though they are benign.^13,14^ Furthermore, AML with invading the renal vein or IVC thrombus, irrespective of size, should be surgically removed even if it is asymptomatic.^8,14^ Radiofrequency ablation or cryoablation may be effective for small, growing AMLs.^13^ For patients with large tumors or a tumor thrombus in the renal vein and IVC, a nephron-sparing surgery or radical nephrectomy plus caval thrombectomy may be performed. For patients with acute, life-threatening hemorrhage, the preferred therapy is selective transarterial embolization.^15^ Before the nephrectomy, to decrease the risk of pulmonary embolism the diagnostically venacavography should identify the level of tumor thrombus and IVC filter must be placed in the suprarenal position. Implanting temporary filter can prevent fatal pulmonary complication and avoid potential side effects of permanent filter such as extremity edema, infection, and organ dysfunction.^6^

It should be noted that AML has the potential to extend into the renal vein, IVC, and right atrium, although it is classified as a benign tumor. There have only a few cases reported so far of pulmonary embolism in patients with renal AML.^5,8^ In this case report, we present a renal AML with extension into renal vein and IVC that was associated with diagnosis of a pulmonary embolism. AML with such characteristics should be managed cautiously with prompt surgical intervention. Before the surgical treatment IVC filter should be implanted if necessary to prevent pulmonary complications.
